# Efficient UAV-based mobile edge computing using differential evolution and ant colony optimization

**DOI:** 10.7717/peerj-cs.870

**Published:** 2022-02-04

**Authors:** Mohamed H. Mousa, Mohamed K. Hussein

**Affiliations:** 1 Department of Information Technology, College of Computer Science at AlKamil, University of Jeddah, Jeddah, Saudi Arabia; 2 Department of Computer Science, Faculty of Computers and Informatics, Suez Canal University, Ismailia, Egypt

**Keywords:** Internet of things, Mobile edge computing, Computation offloading, Differential evolution, Ant colony optimization, Particle swarm optimization

## Abstract

Internet of Things (IoT) tasks are offloaded to servers located at the edge network for improving the power consumption of IoT devices and the execution times of tasks. However, deploying edge servers could be difficult or even impossible in hostile terrain or emergency areas where the network is down. Therefore, edge servers are mounted on unmanned aerial vehicles (UAVs) to support task offloading in such scenarios. However, the challenge is that the UAV has limited energy, and IoT tasks are delay-sensitive. In this paper, a UAV-based offloading strategy is proposed where first, the IoT devices are dynamically clustered considering the limited energy of UAVs, and task delays, and second, the UAV hovers over each cluster head to process the offloaded tasks. The optimization problem of dynamically determining the optimal number of clusters, specifying the member tasks of each cluster, is modeled as a mixed-integer, nonlinear constraint optimization. A discrete differential evolution (DDE) algorithm with new mutation and crossover operators is proposed for the formulated optimization problem, and compared with the particle swarm optimization (PSO) and genetic algorithm (GA) meta-heuristics. Further, the ant colony optimization (ACO) algorithm is employed to identify the shortest path over the cluster heads for the UAV to traverse. The simulation results validate the effectiveness of the proposed offloading strategy in terms of tasks delays and UAV energy consumption.

## Introduction

Mobile edge computing (MEC) has emerged as a promising foundation for providing quality of service (QoS) requirements for the Internet of Things (IoT) and mobile devices to overcome the limited resource capabilities of these devices in terms of processing cycles and energy (Hussein & Mousa, 2020). MEC is an edge network of servers supported by a backend layer of cloud computing located near the IoT and mobile devices network. Oﬄoading computational tasks of the IoT and mobile devices to the edge servers improves execution delay and power consumption of devices by taking advantage of low bandwidth and high latency (Wang et al., 2017). However, there are certain locations where deploying edge servers could be diﬃcult or even impossible, such as hostile terrain, deserts, mountains, underwater, wilderness areas, and disaster areas where the network is down (Mohamed et al., 2017; Cheng et al., 2018).

Unmanned aerial vehicles (UAVs) have significantly advanced in both technological aspects and cost aspects and have shown prominent success in distinct applications, including military, traﬃc control, farming and wilderness monitoring applications (Bejaoui, Park & Alouini, 2020). This outstanding success is motivated by the agility, mobility, and cost-eﬀective deployment of UAVs (Zhang et al., 2019). UAVs can be employed in MEC architecture in two distinct ways: (1) UAV-assisted communication MEC architectures, and (2) UAV-based computation oﬄoading MEC architecture. In the former approach, UAVs serve as relays for distant ground base stations, allowing fast, flexible, and cost-eﬀective network coverage for IoT devices (Wang et al., 2019; Fu et al., 2020). In the latter approach, an edge server is mounted on a UAV for processing oﬄoaded computation tasks of ground mobile devices. This integration of a UAV with an MEC network and the short distance line-of-sight (LoS) wireless communication between the UAV and wireless devices improves the QoS requirements of mobile applications in terms of delay sensitivity as well as the energy consumption of the wireless devices (Mao et al., 2017; Zhou et al., 2020). However, UAVs suﬀer from limited energy and computation capacity constraints, which aﬀect the delay of oﬄoaded tasks. These constraints represent a major challenge that needs to be addressed in UAV-based computation oﬄoading MEC architectures.

In this paper, an intelligent, UAV-based, computation oﬄoading strategy is investigated using evolutionary metaheuristics, where a single UAV-based edge server is utilized to provide computation oﬄoading service to ground IoT and mobile devices. The proposed intelligent architecture aims to minimize the optimization objective of UAV energy and task delays. Two key challenges need to be addressed: (1) the deployment of the UAV, and (2) the shortest trajectory path for the UAV over the set of deployments coordinates. The IoT devices are partitioned into clusters, and the UAV hovers over each cluster head to process the oﬄoaded tasks of the cluster members. A UAV shortest trajectory over the cluster heads is optimized.

UAV energy, deployment, UAV trajectory, and task delays are key challenges in several UAV-based, MEC architecture (Wu et al., 2019; Li et al., 2020a; Wang et al., 2021; Fu et al., 2021). However, approaches such as greedy search and exhaustive search cannot be applied to such optimization problems because there are many discrete and continuous decision parameters, including number, location of deployment, the oﬄoading decision in each deployment, and the shortest trajectory path. Consequently, the complexity of these approaches is extensive and the execution time is extremely high. Further, deep learning approaches require training data, which may not be feasible in a mobile and stochastic environment. Therefore, the optimization problem is divided into two subproblem in order to find a near optimal solution in real-time for such nondeterministic polynomial-time (NP)-hard problems in an acceptable complexity and time. The first subproblem is clustering the IoT devices into groups where the UAV hovers over each group for processing the oﬄoaded tasks of each group members. This problem is an NP-hard complex optimization problem, and evolutionary algorithms can be applied to find a near optimal solution in an acceptable computation time (Hu et al., 2019, 2020; Wu et al., 2020). Therefore, we investigate the use of evolutionary meta-heuristics, including diﬀerential evolution (DE), particle swarm optimization (PSO), and genetic algorithms (GAs), to obtain a near-optimal solution for the clustering problem considering energy and time. The second subproblem is the determination of the shortest UAV trajectory over the clusters head considering the time and energy of the UAV flying. The contributions of this paper are presented as follows:

- The distribution of mobile devices in a specific area is non-uniform. Therefore, partitioning and load balancing oﬄoaded computing tasks into a set of regions severely impacts the performance of the oﬄoading system. Performance degradation may occur, as some regions may become heavily loaded with requests while other regions are lightly loaded with requests. As a result, the waiting time increases in the heavily loaded regions, and some oﬄoading requests could fail. In addition, the UAV energy may be lost if a large number of clusters is set. Therefore, the UAV deployment optimization problem is formulated as mixed-integer, nonlinear constraint optimization that is aimed at minimizing the number of clusters while considering the energy consumption of the UAV and the delays of the oﬄoaded tasks.
- Two discrete diﬀerential meta-heuristics are proposed with mutation and crossover operators, namely, discrete DE (DDE) and discrete PSO (DPSO), for the formulated optimization problem. The proposed DDE algorithm is proposed to obtain a near-optimal solution for the number of clusters, members of each cluster and load balancing tasks in the determined clusters.
- The energy of the UAV is limited, and the UAV follows a determined trajectory over diﬀerent regions to process the oﬄoaded computational tasks and to return the results. Therefore, the UAV trajectory should be optimized to address the energy limitations considering the communication, computation, and mechanical operations of flying and hovering. The partitioned regions are employed as an initial population for an ant colony optimization (ACO) algorithm to obtain a near-optimal solution for minimizing the UAV trajectory.
- Several extensive experiments are performed with the proposed strategy. The evaluations show that the proposed oﬄoading system is eﬀectively capable of significantly improving the task delay and energy consumption compared with a discrete particle swarm optimization (DPSO) and the genetic algorithm (GA).

The remainder of the paper is organized as follows: “Related Work” introduces the related work of UAV-assisted, mobile edge environments. “UAV-Based Oﬄoading System Model and Problem Formulation” presents a system model and a formulation of the optimization problem, while “Proposed Meta-Heuristics” outlines the proposed optimization using DDE, DPSO, and ACO. In “Experimental Results”, the experimental results of the proposed strategy are presented and evaluated, followed by a summary and conclusion of the paper with a discussion of future research directions in “Conclusion and Future Work”.

## Related work

The MEC architecture is a successful solution for the limited computational resources in terms of computation and energy, and thus, the performance of the oﬄoaded tasks are improved by taking advantage of the low latency and high bandwidth of the edge servers (Yu, 2016; Zakaryia, Ahmed & Hussein, 2020). UAVs have the advantages of agility, mobility and fast deployment and can provide on-demand communication and computation services when mounted with communication equipment and MEC servers with a LoS advantage, which will produce better transmission rates with reduced energy consumption of the mobile devices. However, the limitation of the energy of the UAV aﬀects the communication and computational capacity, as well as the service time oﬀered to the ground mobile equipment (Nguyen et al., 2020). Therefore, diﬀerent research eﬀorts are conducted for the key challenges based on the diﬀerent configurations, optimization objectives, and underlying constrains.

In Hu et al. (2019a), the UAV trajectory and ratio of oﬄoading tasks are jointly optimized with the aim of minimizing the sum of the maximum delay of all users in diﬀerent time slots, as statistically specified. In Wang et al. (2020a), the task oﬄoading decisions are reached based on the objective of minimizing oﬄoading delay and energy consumption considering bandwidth, size of the data, and power consumption. The oﬄoading decision for each mobile task includes oﬄoading to the UAV or oﬄoading to the ground MEC through the UAV. However, the study does not consider the optimization of UAV trajectories. In Tang et al. (2020), a partial oﬄoading UAV-based MEC system that is aimed at maximizing the number of oﬄoading tasks is proposed. The oﬄoading problem is formulated as a mixed-integer, nonlinear programming problem and is solved using block coordinate descent (BCD) and a convex optimization technique. However, the mobility of the UAV is not considered in the proposed model. In Yang et al. (2020), DE with deep reinforcement learning is selected for the deployment of multi-UAVs with the aim of minimizing the oﬄoaded task delays and load balancing UAVs loads. In Li et al. (2020a), a successive complex optimization technique is utilized to jointly optimize the UAV trajectory and computational load allocation with the aim of minimizing UAV energy. However, the proposed system does not consider the distribution of the mobile devices on the ground area or the time requirements of the oﬄoaded tasks. In Wang et al. (2020b), a two-layer optimization method is proposed to jointly optimize multi-UAV deployment and mobile task allocation and to minimize UAV energy consumption. The upper layer of the optimization method uses a DE algorithm that minimizes the number of UAVs, and in the lower layer, a greedy algorithm is proposed for identifying an optimal solution whether tasks are oﬄoaded or processed locally. In Chen et al. (2020), an intelligent task oﬄoading system is proposed. The proposed system intelligently perceives the network environment and makes oﬄoading decisions using a Monte Carlo tree search. Furthermore, a deep neural network is applied to optimize the search based on the latency delay. However, the proposed oﬄoading strategy requires training data, a prediction model for the channel state, and time for the self-learning process. In Hu et al. (2019b), a UAV is connected with a cellular base station as either a relay to the base station or as a computing server for the oﬄoaded computation tasks of the mobile equipment. A greedy search based on nonconvex optimization is proposed with the aim of minimizing the weighted sum energy of the UAV and the mobile devices by jointly optimizing the computational resource scheduling, allocation of bandwidth resources, and trajectory of the UAV. In Li et al. (2020b), an energy-eﬃcient, UAV-based, oﬄoading architecture that optimizes the bit allocations in diﬀerent regions of user tasks and the trajectory of the UAV using successive approximation is proposed. However, the proposed scheme does not address how to partition the fixed clustered slots, and further, the time delay is not considered in the proposed scheme. In Zhan et al. (2020), a joint optimization of computation oﬄoading, resource allocation, and UAV trajectory is proposed with the aim of minimizing the energy consumption of the UAV. In Guo & Liu (2020), the UAV energy is optimized considering the transmitted bits in both the uplink and downlink and the UAV trajectory using a greedy search based on the successive convex approximation. In Yu et al. (2020), an alternative optimization algorithm based on successive convex approximation (SCA) is proposed to minimize the weighted sum of the service delay of the IoT tasks by jointly optimizing computing oﬄoading, resource allocation and trajectory.

In summary, approaches such as greedy search and exhaustive search cannot be applied to such an optimization problem because there are many discrete and continuous decision parameters, including the number, location of deployment, oﬄoading decision in each deployment, and shortest trajectory path. Therefore, most research uses heuristic approaches with fixed partitions; these approaches are not scalable because their complexity is extensive. Further, deep learning approaches, such as Lan et al. (2019), Wang et al. (2021), requires training data which may not feasible in the mobile and stochastic environment. Therefore, we opt to use evolutionary meta-heuristics, including DE, PSO, and GAs, to obtain a near-optimal solution for such NP-hard problems in an acceptable time. The proposed UAV-based oﬄoading system determines the optimal number of clusters and specifies the member tasks of each cluster which are permitted to oﬄoad when the UAV hover the cluster head considering the UAV energy and task delay constraints.

## Uav-based offloading system model and problem formulation

A UAV-based oﬄoading system consists of two layers. The first layer, the ground layer, is the IoT and mobile device layer, which has *N* stationary wireless devices *S* = {*s*_1_, *s*_2_, …, *s*_*N*_} on the ground. The wireless devices oﬄoad their computations to the edge layer to accelerate computations and to optimize energy consumption of the IoT devices. The second layer is the flying MEC layer, which consists of a single UAV that is supported with an edge server that oﬀers computation oﬄoading services for the ground layer with minimum latency. The UAV flies at a steady altitude *H* > 0. The UAV has the communication coverage radius *R*_*c*_. The UAV flies according to a specified trajectory over a set of *K* regions starting from a specific predetermined starting point. The UAV hovers over each region *k* to process the oﬄoaded tasks that are scheduled in this region, and the UAV returns to the starting point by the end of the trajectory cycle.

The coordinates of each *s*_*i*_ device location in the ground layer are known in advance and are given by *s*_*i*_ = {*x*_*i*_, *y*_*i*_}. The coordinates of the UAV position in region *k* are given by *u*_*k*_ = {*x*_*k*_, *y*_*k*_}, and the distance between the UAV and *s*_*i*_ is calculated using the Euclidean distance:

(1) $$dis{t_{ik}} = {[{({x_k} - {x_i})^2} + {({y_k} - {y_i})^2} + {H^2}]^{{1 \over 2}}}$$

where device *s*_*i*_ should be in the cover radius *R*_*c*_ of the UAV.

(2) $$dis{t_{ik}} < {R_c}$$

Device *s*_*i*_ is requesting to oﬄoad task ${t_i} = \{ t_i^c,t_i^\tau ,t_i^{data}\}$, where $t_i^c$, $t_i^\tau$, and $t_i^{data}$ are the required computing cycles, task delay, and data size, respectively. In the proposed UAV-based oﬄoading system, the set of ground devices *s*_*i*_∀*i* ∈ *N* are partitioned into *K* regions, where the UAV hovers over each region to process the oﬄoaded tasks, and *δ*_*ik*_ = 1 when mobile device *s*_*i*_ oﬄoads its task *t*_*i*_ to be processed when the UAV hovers over region *k*. The following subsection presents the system formulation, followed by a formulation of the objective function. The key abbreviations used in the paper are listed in Table 1. The following subsections present the formulations for the computation, the communication, and the power consumption model for the optimization problem.

**Table 1:** List of abbreviations.

Abbreviation	Definition
UAV	Unmanned arial vehicle
IoT	Internet of Things
QoS	Quality of service
MEC	Mobile edge computing
DE	Diﬀerential evolution
DDE	Discrete differential evolution
PSO	Particle swarm optimization
DPSO	Discrete particle swarm optimization
GA	Genetic algorithm

### Communication model

Assume that the UAV has a ground coverage range with a radius of *R*_*c*_. Similar to Bejaoui, Park & Alouini (2020), the time-varying channel gain between a ground IoT device *s*_*i*_ within partition *k* to the UAV *via* orthogonal frequency division multiplex access (OFDMA) is calculated using Eq. (3).

(3) $${h_{ik}} = \displaystyle{{{\rho _0}} \over {dist_{ik}^2}}$$

where *ρ*_0_ is the received power at a reference distance of 1 m.

The transmission rate between sensor *i* and the UAV in region *k* at position *u*_*k*_ = {*x*_*k*_, *y*_*k*_} is calculated as (He et al., 2018) using Eq. (4).

(4) $${r_{ik}} = B{\log _2}(1 + \displaystyle{{{P_i}{h_{ik}}} \over {{N_0}}})$$

where *B* is the bandwidth of the uplink channel, *P*_*i*_ is the maximum transmit power of the wireless device *s*_*i*_, and *N*_0_ is the channel noise.

The transmission time $T_{ik}^{trans}$ for oﬄoading a task from the *i*^*th*^ IoT device to the UAV at region *k* is calculated using Eq. (5).

(5) $$T_{ik}^{trans} = \displaystyle{{t_i^{data}} \over {{r_{ik}}}}$$

The total transmission time at region *k* with position {*x*_*k*_, *y*_*k*_} is calculated using Eq. (6).

(6) $$T_k^{trans}({x_k},{y_k},{\delta _{ik}}_{i = 1}^N) = \sum\limits_{i = 1}^N {{\delta _{ik}}T_{ik}^{trans}}$$

where *δ*_*ik*_ represents the oﬄoading decision for task *t*_*i*_ in region *k* with coordinates {*x*_*k*_, *y*_*k*_}, where *δ*_*ik*_ = 1 means that the task is processed when the UAV hovers over region *k*.

### 3.2 Computation model

The computation time $T_{ik}^{comp}$ for processing task *t*_*i*_ at the UAV is calculated using Eq. (7).

(7) $$T_{ik}^{comp} = \displaystyle{{t_i^c} \over {{f^u}}}$$

where *f*^*u*^ is the processing capacity of the UAV.

Assume that the edge server executes the oﬄoaded tasks in a queue ordered by first come first served. The overall computation time in region *k* when the UAV hovers at position {*x*_*k*_, *y*_*k*_} is calculated using the following equation:

(8) $$T_k^{comp}({x_k},{y_k},{\delta _{ik}}_{i = 1}^N) = \sum\limits_{i = 1}^N {{\delta _{ik}}T_{ik}^{comp}}$$

The total time for the UAV in region *k* is the total oﬄoading delay, which is calculated as the sum of the transmission delay and computation delay using the following equation:

(9) $${T_k}({x_k},{y_k},{\delta _{ik}}_{i = 1}^N) = T_k^{trans}({x_k},{y_k},{\delta _{ik}}_{i = 1}^N) + T_k^{comp}({x_k},{y_k},{\delta _{ik}}_{i = 1}^N)$$

### Energy consumption model

The energy model which is used in this study follows the energy model described in Bejaoui, Park & Alouini (2020) for a fixed wing UAV. The UAV works under limited charged energy capacity, and the propulsion energy consumption is much higher than computation and communication energy (Wang et al., 2020b). The adopted model is simple where the energy is computed using a constant coeﬃcient that depends on the architecture. For example, the hovering energy is calculated by multiplying a hovering energy coeﬃcient by the hovering time. The total UAV energy consumption is a result of several operations, including (1) wireless communication with IoT devices to receive oﬄoaded tasks and to return results, (2) computation of the oﬄoaded tasks, and (3) propulsion energy consumption of flying and hovering. The transmission energy consumption at region *k* is calculated using Eq. (10).

(10) $$E_k^{trans}({x_k},{y_k},{\delta _{ik}}_{i = 1}^N) = {\kappa _1}\sum\limits_{i = 1}^N {{\delta _{ik}}t_i^{data}}$$

where *κ*_1_ is an energy factor for wireless communication.

The computation energy consumption in region *k* is calculated using Eq. (11).

(11) $$E_k^{comp}({x_k},{y_k},{\delta _{ik}}_{i = 1}^N) = {\kappa _2}\sum\limits_{i = 1}^N {{\delta _{ik}}t_i^c{f^u}^2}$$

where *κ*_2_ is an energy factor for computation processing.

Following the propulsion energy model of the UAV proposed in reference to the propulsion model proposed in Wu et al. (2019), the propulsion energy consumption for hovering in region *k* is calculated using Eq. (12).

(12) $$E_k^h({x_k},{y_k},{\delta _{ik}}_{i = 1}^N) = \gamma {T_k}({x_k},{y_k},{\delta _{ik}}_{i = 1}^N)$$

where *γ* is an energy factor for hovering and ${T_k}({x_k},{y_k},{\delta _{ik}}_{i = 1}^N)$ is the time in which the UAV hovers in region *k* to process the oﬄoaded tasks, calculated using Eq. (9).

The propulsion energy consumption for flying to region *k* is calculated using Eq. (13).

(13) $$E_k^{fly}({x_k},{y_k}) = \Gamma \displaystyle{{||{u_k} - {u_{k - 1}}|{|^2}} \over \mu }$$

where Γ is an energy factor for flying and *μ* is the UAV speed between the coordinates of regions *u*_*k*−1_ and *u*_*k*_.

The total energy consumption of the UAV is the sum of the resulting transmission energy consumption, resulting computing energy, and propulsion energy consumption for hovering and flying. Therefore, the total energy consumption for region *k* is calculated using the following equation:

(14) $$\eqalign{
  & {E_k}({x_k},{y_k},{\delta _{ik}}_{i = 1}^N) = E_k^{trans}({x_k},{y_k},{\delta _{ik}}_{i = 1}^N) + E_k^{comp}({x_k},{y_k},{\delta _{ik}}_{i = 1}^N) + E_k^{fly}({x_k},{y_k})  \cr 
  & \quad \quad \quad \quad \quad \quad \quad \quad  + E_k^h({x_k},{y_k},{\delta _{ik}}_{i = 1}^N) \cr} $$

### UAV deployment and optimization

The aim of the study is to design an intelligent UAV-based oﬄoading strategy where the IoT devices are dynamically clustered considering the number of clusters *K*, members of the clusters, and total energy. The cluster members can be measured using the maximum number of members in the clusters $\dot m({\delta _{ik}})$ and the sum of the distances between the members and their cluster centers $\dot D({\delta _{ik}})$ according to the following Equations:

(15) $$\dot m(K,{\delta _{ik}}_{i = 1}^N) = \max _{k = 1}^K \bigg(\sum\limits_{i = 1}^N {{\delta _{ik}}} \bigg)$$

(16) $$\dot D(K,{\delta _{ik}}_{i = 1}^N) = \sum\limits_{k = 1}^K {\sum\limits_{i = 1}^N {{\delta _{ik}}} } dis{t_{ik}}$$

The total energy *E* over *K* clusters are calculated using the following Equation:

(17) $$E(K,{\delta _{ik}}_{i = 1}^N) = \sum\limits_{k = 1}^K {{E_k}} ({x_k},{y_k},{\delta _{ik}}_{i = 1}^N)$$

The optimization problem of dynamically determining the optimal number of clusters, specifying the member tasks of each cluster, is modeled as mixed-integer, nonlinear constraint optimization and is formulated as follows:

(18) $${\bf Minimize}\!:\quad \;\;\;F = \displaystyle{{K\,E(K,{\delta _{ik}}_{i = 1}^N)} \over {\dot m(K,{\delta _{ik}}_{i = 1}^N)\,\dot D(K,{\delta _{ik}}_{i = 1}^N)}}$$

(19) $${\bf Minimize}\!:\quad \;\;\;F = \displaystyle{{K\,\sum\limits_{k = 1}^K {{E_k}} ({x_k},{y_k},{\delta _{ik}}_{i = 1}^N)} \over {\max _{k = 1}^K(\sum\limits_{i = 1}^N {{\delta _{ik}}} )\,\sum\limits_{k = 1}^K {\sum\limits_{i = 1}^N {{\delta _{ik}}} } dis{t_{ik}}}}$$

Subject to the following constraints:

(20) $${C_1} = R_c^2 - (dist_{ik}^2 + {H^2}) \le 0,\forall \,i = \{ 1,...,N\} ,k = \{ 1,...,K\}$$

(21) $${C_2} = \sum\limits_{k = 1}^K {{T_k}} ({x_k},{y_k},{\delta _{ik}}_{i = 1}^N) - {T_u} < 0$$

(22) $${C_3} = \sum\limits_{k = 1}^K {{E_k}} ({x_k},{y_k},{\delta _{ik}}_{i = 1}^N) - {E_u} < 0$$

(23) $${C_4} = \sum\limits_{k = 1}^K {{\delta _{ik}} = 1\;\forall \,i = \{ 1,...,N\} }$$

*C*_1_ ensures that the determined cluster members fall within the communication radius of the cluster. Constraint *C*_2_ states that the overall time required for the transmission and computations over all regions is less than the maximum flaying time of the UAV, *T*_*u*_. Constraint *C*_3_ states that the overall energy required for the UAV over all regions, including communication and computation as well as hovering, is less than the maximum energy of the UAV, *E*_*u*_. *C*_4_ states that the oﬄoading decision of each task is allocated to only one oﬄoading region. To address the constraints, a penalty value is added to the optimization function for each violated constraint using the following Equation:

(24) $$\dot F = F + \lambda \sum\limits_{{c_k}} {C_k^2}$$

where *λ* is a penalty value. $C_k^2$ is the value of the violated constraint *k* squared.

## Proposed meta-heuristics

UAV energy, deployment, UAV trajectory, and task delays are key challenges in a UAV-based MEC architecture. Obtaining the optimal solution for this problem using greedy search and exhaustive search optimization requires very high complexity algorithm with extremely high execution time. Also, the optimization problems involves many discrete and continuous decision parameters, including number, location of deployment, the oﬄoading decision in each deployment, and the shortest trajectory path. Further, deep learning approaches require training data, which may not be feasible in a mobile and stochastic environment. Therefore, the optimization problem is divided into two subproblem in order to find a near optimal solution in real-time for such complex (NP)-hard optimization problem in an acceptable complexity and time. The first subproblem is the clustering the IoT devices into groups where the UAV hovers over each group for processing the oﬄoaded tasks of each group members. In this section, we investigate the use of evolutionary meta-heuristics, including diﬀerential evolution (DE), particle swarm optimization (PSO), and genetic algorithms (GAs), to obtain a near-optimal solution for the clustering problem considering energy and time. Also, the second subproblem is the determination of the shortest UAV trajectory over the clusters head considering the time and energy of the UAV flying. Also, in this section, we investigate the use of the ACO optimization algorithm to identify the shorest trajectory that satisfy the time and energy constrains. Evolutionary and nature-inspire optimization meta-heuristics, such as DE, PSO, GA, and ACO, have the potential to obtain a near-optimal solution in an acceptable time (Islambouli & Sharafeddine, 2019). The following subsections describe the use of diﬀerent meta-heuristics, namely DE, PSO, and ACO, to optimize the UAV-based MEC oﬄoading system.

### DE meta-heuristic

DE is a population-based stochastic search process. DE has a few control parameters and strong search capabilities compared to most search meta-heuristics and is able to solve complex, nonlinear optimization functions (Deng et al., 2021). The DE simulates biological evolution in nature using mutation, crossover, and selection operations. The process iterates for a fixed number of iterations, and the population is continuously updated. The process starts with initialization of the population, where *Np* individuals, *X*_*i*_, *i*∈ [1 *Np*], are randomly initialized. Each individual represents a possible solution to the underlying problem. The DE mutation step generates new candidate individuals *V*_*i*_ to provide diverse mutants for better exploration of the search space. The most common mutation strategies are:

(25) $${\bf DE}/{\bf rand}/{\bf 1}\quad \;\;\;V = {X_{r1}} + F.({X_{r2}} - {X_{r3}})$$

(26) $${\bf DE}/{\bf best}/{\bf 1}\quad \;\;\;V = {X_{best}} + F.({X_{r1}} - {X_{r2}})$$

where *r*1, *r*2, and *r*3 are randomly generated integers numbers ∈ [1 *Np*]. *F* is a positive factor to scale diﬀerence individuals. *X*_*best*_ is the best individual with the best fitness value in the population.

In the crossover operator, the target individual *X*_*i*_ is combined with mutant individual *V*_*i*_, which resulted from the mutation operation, to produce trial individual *U*_*i*_ according to probability *cr* using the following Equation:

(27) $${U_{ij}} = \left\{ {\matrix{ {{V_{ij}},} \hfill  {{\rm if}\;rand \le cr\;{\rm or}\;j = jrand} \hfill \cr {{X_{ij}},} \hfill  {{\rm otherwise}} \hfill \cr } } \right.$$

where *jrand* is a random integer from the dimension of the search space.

In the selection process, a greedy selection is applied to the trial and corresponding individual from the previous generation. The objective function value of the trial individual *f*(*U*_*i*_) is compared with the objective function value of the corresponding target vector *f*(*X*_*i*_), and the individual with the least function value will survive to the next generation.

(28) $${X_i} = \left\{ {\matrix{ {{U_i},} \hfill  {{\rm if}\;f({U_i}) < f({X_i})} \hfill \cr {{X_i},} \hfill  {{\rm otherwise}} \hfill \cr } } \right.$$

#### Proposed DDE algorithm

The first step in the proposed algorithm is the encoding of the individual solution. Each individual *X*_*i*_ is composed of *N* genes, representing the IoT devices. Each gene will have an ID of the device, which will be the center of the cluster. The center of each cluster represents the cluster head where certain IoT devices belongs to, in which the members only oﬄoad their computation tasks once the UAV hoovers over that cluster head. For example, *X*_1_ = {2, 2, 2, 4, 4, 4} represents an individual solution for six IoT devices, and there are two clusters. The first cluster contains three devices 1, 2, and 3, and the second cluster contains three devices 4, 5, and 6. Devices {2, 4} are the centers of the clusters. This section shows that the cluster heads and their members are optimized by applying the proposed DDE algorithm using the objective function shown in Eq. (24) which considers the total time, energy, number of clusters, cluster members, and the distances between the members in the clusters.

The DE algorithm is designed to solve continuous optimization problems. Therefore, the algorithm must be converted to output a discrete value. A discrete diﬀerential evolution (DDE) is designed using mutation and crossover operators. Algorithm 1 shows the detailed steps of the proposed DDE algorithm. The first step of the algorithm is the initialization of the parameters followed by the random initialization of the population individuals. After that, the mutation step using Eq. (25), is changed to the following Equation:

**Algorithm 1:** Proposed DDE algorithm.

**Result:** *bestFitness*, *X*_*best*_
Initialize *N*, *N*_*p*_, *N*_*iter*_, *cr*, and *F*
Random initialize *X*_*i*_ with random clustering IDs
Calculate the fitness of each possible solution *X*_*i*_ using an optimization function with penalty, Eq. (24)
Set *bestFitness* = *min*(*X*)
Set *X*_*best*_ for the individual with the minimum fitness
**for** *each iter in N*_*iter*_ **do**
**for** *each X*_*i*_ *in N*_*p*_ **do**
Apply the mutation operation, and calculate the mutated individual *V*_*i*_(*t*) using Eq. (29)
Apply the crossover operation, and calculate the target individual *U*_*i*_(*t*) using Eq. (27)
Apply the greedy selection, calculate the new population *X*_*i*_(*t* + 1) using Eq. (28)
** end**
Calculate the fitness of each possible solution in the new population
**if** *bestFitness* > *best fitness of the new population* **then**
update *X*_*best*_ and *bestFitness*
** end**
**end**

(29) $$V = {f_3}({X_{best}},{f_2}(F \oplus {f_1}(X),{X_{r1}}))$$

where *f*_1_ represents a mutation operation on individual *X* with probability *F*. For each gene in the individual, the random number *r* ∈ [0 1] is generated, and the gene is perturbed with a random value while *r* < *F*. A single point crossover operator, *f*_2_, is applied to the resulting perturbed individual with a random individual from the population. A uniform crossover, *f*_3_, is applied to the result of the single point crossover with the best solution found, *X*_*best*_. A greedy selection is applied to the crossover operators *f*_2_ and *f*_3_. The last step involves the crossover and the greedy selection operations described using Eqs. (27) and (28) are applied.

### PSO meta-heuristic

The PSO is a stochastic, population-based, search meta-heuristic that simulates the social behavior of a swarm of birds searching for food. Each particle, an individual bird in the swarm, searches for food in the search space to identify the best food source based on the particle position, particle best found solution, and swarm best found global solution (Zakaryia, Ahmed & Hussein, 2020). Each individual particle represents a possible solution in the search space of the problem under observation. In the iterative search process of PSO, each individual particle *X*_*i*_ updates its value *X*_*i*_(*t* + 1) by updating its corresponding velocity *V*_*i*_(*t* + 1) using the following Eqs. (30) and (31):

(30) $${V_i}(t + 1) = \omega {V_i}(t) + {C_1}\alpha \,(X{l_i} - {X_i}) + {C_2}\beta \,(Xg - {X_i}(t))$$

(31) $${X_i}(t + 1) = {X_i}(t) + {V_i}(t + 1)$$

where *V*_*i*_ and *X*_*i*_ are the velocity position and particle position, respectively, of individual *i*, and *t* is the iteration number. *Xl*_*i*_ is the best position of individual *X*_*i*_, and *Xg* is the global best position for all individuals. *ω* is the inertia weight. *C*_1_ and *C*_2_ are the cognitive coeﬃcient and social coeﬃcient. *α* and *β* are random numbers ∈*N*[0 1]. PSO is an eﬀective solution for optimization problems in the continuous domain, and a discrete version of the PSO algorithm must be adopted to suit the problem under study (Zakaryia, Ahmed & Hussein, 2020). A new Discrete PSO (DPSO) is designed and presented in the following subsection.

### DPSO algorithm

In the DPSO, each individual is updated using mutation and crossover operators similar to the method used in DDE. The same encoding described in the DDE algorithm is employed. The particle position is updated using mutation, and a crossover operator is proposed using the following Equation:

(32) $${X_i} = {C_2} \oplus {f_3}({C_1} \oplus {f_2}(\omega \oplus {f_1}({X_i}),X{l_i}),{X_g})$$

where *ω*⊕*f*_1_(*X*_*i*_) represents a mutation operation applied on individual *X*_*i*_ with probability *w*. *f*_2_ is a single point crossover operator, and *f*_3_ is a uniform crossover operator. *C*_1_ and *C*_2_ serve as probabilities for the single point and uniform crossover operators.

Algorithm 2 shows the steps of the proposed DPSO algorithm. The algorithm starts by initializing the parameters, including the number of devices *N*, population size *N*_*p*_, number of iterations *N*_*iter*_, and mutation and crossover probabilities *ω*, *C*_1_, and *C*_2_. The second step involves initializing the particles with random numbers. In the third step, an evaluation of the population with the objective function is performed using Eq. (24). Steps 5, 6, the local best of each particle and the global best of all particles, are updated. Steps 7–14 are iterated for *N*_*iter*_ iterations. In each iteration, each particle is updated with mutation and crossover operators using Eq. (32), and a greedy selection is applied to the new individuals. The fitness of each particle in the new population is updated. The local best and global best are updated.

**Algorithm 2:** Proposed DPSO algorithm.

**Result:** *bestFitness*, *X*_*best*_
Initialize *N*, *N*_*p*_, *N*_*iter*_, *ω*, *C*_1_ and *C*_2_
Randomly initialize *X*_*i*_ and *V*_*i*_ with random clustering IDs
Calculate the fitness of each possible solution *X*_*i*_ using an optimization function with penalty, Eq. (24)
Update the best position *Xl*_*i*_ for each individual
Update the global position *X*_*g*_ for all individuals
Set *X*_*g*_ = *min*(*Xl*)
**for** *each iter in N*_*iter*_ **do**
**for** *each X*_*i*_ *in N*_*p*_ **do**
Apply mutation and crossover operations using Eq. (32)
**end**
Calculate the fitness of each possible solution in the new population
Update *Xl*_*i*_ for each individual
Update *X*_*g*_ = *min*(*Xl*)
**end**

### UAV shortest trajectory using ACO

The solution of the proposed DDE algorithm identifies a set of clusters with their members. The following algorithm optimizes the shortest path among the cluster centers for the UAV to traverse using ACO. Compared to the classical algorithms of path planning, the ACO has the capability to deal with complex, dynamically changing environments, and several research studies have shown that ACO can obtain a near optimal path solution for the shortest path problem in an acceptable computational time for small and large-scale networks (Ouyang et al., 2021). Also, the ACO meta-heuristic has the advantage that it can escape the local optima and finding the global optima (Puris, Bello & Herrera, 2010).

ACO is a meta-heuristic search algorithm that is based on the behavior of a group of ants in the process of searching for food. The behavior of the group optimizes an objective through a feedback strategy that ants use to identify the optimal path between the colony and the food source. Ants start leaving the colony and select random path searching of food sources. Ants communicate with each other by releasing a chemical, referred to as a pheromone, with a quantity proportional to the quantity and quality of the discovered food. This pheromone is considered an evaluation for the optimization objective. Eventually, all ants discover the shortest path by following the path corresponding to the highest pheromone concentration. The ACO meta-heuristic is an eﬃcient search algorithm for NP-hard problems, including traveling salesman, job shop scheduling, and scheduling in mobile edge computing (Hussein, Mousa & Alqarni, 2019).

Given the set of points *K* that represent the cluster head locations. The goal is to identify the shortest path among the points for the UAV to traverse for hovering to process the oﬄoaded tasks. The length of the path from the first cluster head to the last cluster head is considered because the flying time and the flying energy are completely dependent on the length of the trajectory path of the UAV. The following two conditions must be satisfied on the the determined UAV trajectory path:

(33) $${C_1} = T + {T^{fly}} \le {T_u}$$

(34) $${C_2} = E + {E^{fly}} \le {E_u}$$

where *C*_1_ ensures that the total time including communication, computation, and flying, *T*^*fly*^, is less than the total flying time of the UAV *T*_*u*_. Also, constraint *C*_2_ states that the overall energy including computation, communication, hovering and flying is less than the maximum energy of the UAV, *E*_*u*_. *T*^*fly*^ and *E*^*fly*^ are calculated using the following two Equations:

(35) $${T^{fly}} = \displaystyle{{\sum\limits_{i = 1,j = i + 1}^{K - 1} | |{u_i} - {u_j}|{|^2}} \over \mu }$$

(36) $${E^{fly}} = \Gamma \;{T^{fly}}$$

where Γ and *μ* are the flying energy factor and the UAV speed between the clusters head, and *u*_*i*_ is the position of the cluster head *i*.

Initially, all ants are randomly placed on cluster heads. The algorithm iterates for a fixed number of iterations *N*_*iter*_. During each iteration, each ant *m* selects the next cluster head *j* from cluster *i* with probability $P_{ij}^m(t)$, calculated using Eq. (37).

(37) $$P_{ij}^k(t) = \displaystyle{{{{({\tau _{ij}}(t))}^\alpha }{{({\eta _{ij}}(t))}^\beta }} \over {\sum\limits_j {{{({\tau _{ij}}(t))}^\alpha }} {{({\eta _{ij}}(t))}^\beta }}}$$

where *t* is an iteration number, and *α* is a heuristic variable that controls the eﬀect of the pheromone quantity and *β* is a heuristic parameter that specifies the quality of the node selection. *η*_*ij*_(*t*) is a heuristic function that represents the quality of the next node selection; it is calculated using Eq. (38).

(38) $${\eta _{ij}}(t) = \displaystyle{Q \over {d(i,j)}}$$

where *Q* is a constant and *d*(*i*, *j*) is the Euclidean distance between the points *i* and *j*. $\tau _{ij}^m(t)$ represents the pheromone trail quantity for the selected path for an ant *m* in iteration *t*, calculated using Eq. (39).

(39) $$\tau _{ij}^m(t + 1) = (1 - \rho )\tau _{ij}^m(t) + \rho \Delta\tau _{ij}^m(t)$$

where $\Delta\tau _{ij}^m(t) = 1/{L^m}$ and *ρ* is a constant that represents the rate of pheromone evaporation at each step. *L*^*m*^ is the length of the trajectory path determined by ant *m*.

Once all ants have completed their path finding, *i.e*., a complete iteration has been performed, the pheromone trail is updated globally. The global pheromone update is calculated using Eq. (40).

(40) $$\tau _{ij}^m(t + 1) = (1 - {\rho _g})\tau _{ij}^m(t) + {\rho _g}\Delta\tau _{ij}^m(t)$$

where $\Delta\tau _{ij}^m(t) = 1/{L_{best}}$, *L*_*best*_ is the best shortest path discovered, and *ρ*_*g*_ is the global evaporation rate.

Algorithm 3 shows the steps of the proposed ACO-based UAV shortest path among a set of cluster heads. The proposed algorithm starts by initializing the parameter settings *α* and *β*; the local pheromone concentration *Q*; the local pheromone decay parameter *ρ*; the global pheromone decay parameter *ρ*_*g*_; the number of ants *N*_*ants*_; the number of iterations *N*_*iter*_; and the number of cluster heads points *N*_*k*_. Step 2 initializes the pheromone matrix $\tau _{ij}^m(0) = 1/d(i,j)$ for each ant *m*. During each iteration, for each ant, *ant*_*m*_ searches for a complete path journey. All ants are randomly placed on initial points. The selection of the next point *j* is calculated with probability $P_{ij}^m(t)$ using Eq. (37). The selection of the next point is performed using the roulette wheel selection method (Holland, 1992). When an ant finishes a complete journey, the local pheromone trail matrix is updated using Eq. (39). After each iteration, the ACO-based algorithm updates the global pheromone trail matrix $\tau _{ij}^m(t)$ using Eq. (40). The algorithm iterates until the maximum number of iterations is reached.

**Algorithm 3:** The ACO-based UAV trajectory planning algorithm.

**Result:** Shortest path for the UAV to traverse.
Initialize the parameters *α*, *β*, *Q*, *ρ*, *ρ*_*g*_, *N*_*ants*_, *N*_*iter*_, and *N*_*k*_
Create an initial population of ants
Initialize the pheromone matrix $\tau _{ij}^m(0) = 1/d(i,j)$
**While** *iter* ≤ *N*_*iter*_ **do**
Randomly place all ants at the starting nodes
**for** *each ant*_*m*_ **do**
**while** *ant*_*m*_ *has not finished its journey* **do**
**for** *each j in N*_*k*_ ∉ *Journey*^*m*^ **do**
//ADD node *j* to the current journey of the *ant*_*m*_ solution
Calculate $\eta _{ij}^m(t)$ using Eq. (38)
Calculate $P_{ij}^m(t)$ using Eq. (37)
*ant*_*m*_ chooses the cluster head *j* using the roulette method
**end**
**end**
Update $\tau _{ij}^m(t + 1)$ using Eq. (39)
**end**
Compare all ant solutions with the previous best solution and update the best solution *Journeybest*
**if** *the current solution is the best* **then**
Update $\tau _{ij}^m(t + 1)$ using Eq. (40)
**end**
**end**

## Experimental results

**Figure 1:**
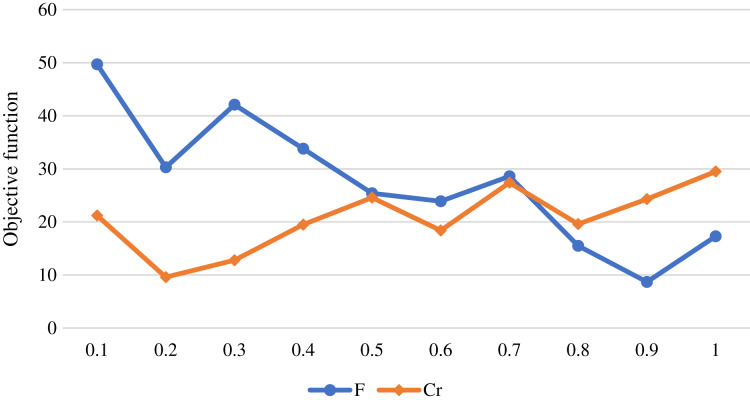
The value of the objective function for diﬀerent values of the DDE algorithm parameters.

This section provides the simulation results that verify the performance of the proposed UAV-based oﬄoading systems using the proposed meta-heuristics. The IoT devices are randomly deployed in a 200 *m* × 200 *m* area with the simulation parameters that are established according to the values described in Table 2. Diﬀerent possible values of the meta-heuristics parameters are tested in the experiments, and the best value that obtained the best results are reported. However, there are many methods for tuning hyperparameters of the meta-heuristics, including grid search, artificial neural network, and bayesian optimization across continuous spaces which can be considered in our future works. The proposed DDE algorithm has two parameters *F* and *Cr*, and Fig. 1 shows the impact of the diﬀerent values of the DDE algorithm parameters on the value of the objective function. The figure shows that the best values that maintain lowest objective function value are F = 0.9 and CR = 0.2. The GA has a crossover value set to 1 and a mutation probability set to 0.05. The DPSO parameters are set to *w* = 0.2, *c*_1_ = 0.9, and *c*_2_ = 0.9. The maximum number of iterations is set to 500. The collected results are based on applying 10 diﬀerent runs, and the average is calculated.

**Table 2:** Parameter settings for the evaluation experiments.

Parameter	Description	Value
*B*	Bandwidth	40 *MHz*
*h*_*i*_	Channel gain	−30 dB
*N*_0_	Noise power	10^−9^ *W*
*κ*_1_	UAV energy communication coeﬃcient	10^−18^
*κ*_2_	UAV energy computation coeﬃcient	10^−26^
γ	UAV energy hoovering coeﬃcient	10^−10^ *W*
Γ	UAV energy flying coeﬃcient	10^−8^ *W*
*P*_*ik*_	Maximum transmission power	0.4 W
μ	Maximum flying speed of the UAV	20 *m*/*s*
*H*	Flying altitude of the UAV	50 *m*
*t* ^*data*^	Task data size	200 *KB*−3 *MB*
*t* ^*c*^	Required task computing cycles	6 × 10^9^−9 × 10^10^
*f* ^*u*^	CPU-cycle frequency of the UAV	300 *MHz*
*E*_*u*_	Battery storage capacity of the UAV	5 × 10^5^*J*

**Figure 2:**
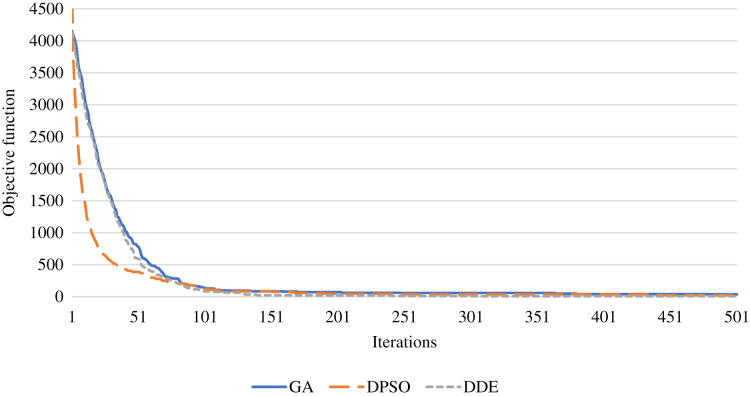
The value of the objective function using the proposed meta-heuristics during the iterations.

To evaluate the convergence of the proposed meta-heuristics in terms of clustering eﬃciency, Fig. 2 shows the value of the objective function of the DDE, DPSO, and GA meta-heuristics during the iterations of a single run. The DDE algorithm is capable of achieving the best convergence compared to DPSO and the GA within the fewer number of iterations. Also, the DPSO with with the mutation and crossover operators can achieve better performance than the GA algorithm.

**Figure 3:**
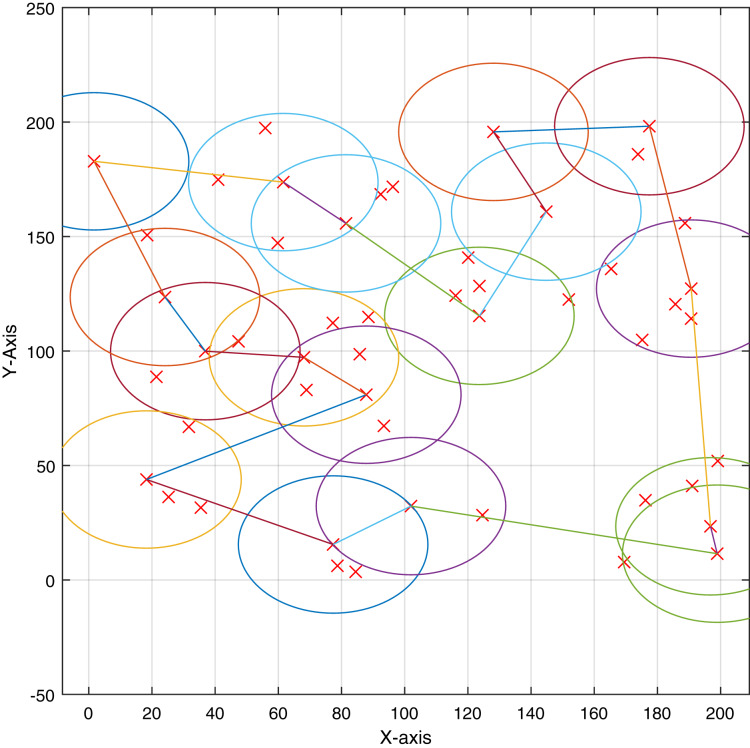
Example of the clustering process using the DDE algorithm for 30 IoT devices.

**Figure 4:**
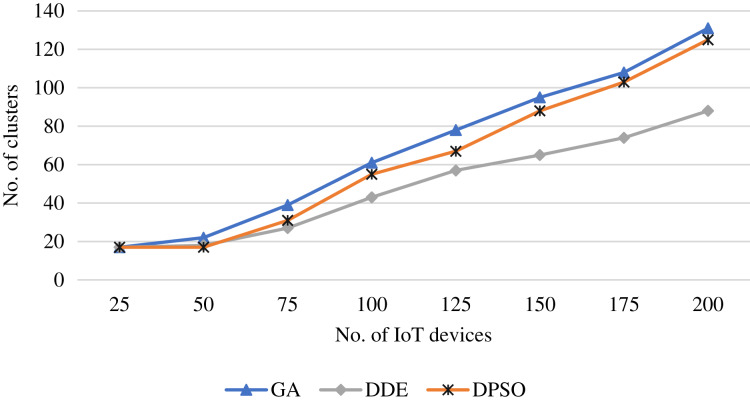
Average number of clusters obtained using the proposed meta-heuristics as increasing the IoT devices.

Figure 3 shows the clusters resulting from the DDE algorithm for 50 IoT devices. The algorithm successfully produced 30 clusters and their members fall within the communication radius of the cluster heads. Further, the shortest path for the UAV is eﬃciently produced using the ACO algorithm. To evaluate the clustering process in terms of the number of clusters, Fig. 4 shows the resulting average number of clusters using the proposed meta-heuristics for diﬀerent runs as increasing the number of IoT devices. The proposed DDE algorithm maintains the lowest number clusters. The DDE algorithm is able to obtain the global optimum compared to the PSO and GA algorithms, and thus, a better clustering is maintained using the DDE algorithm.

**Figure 5:**
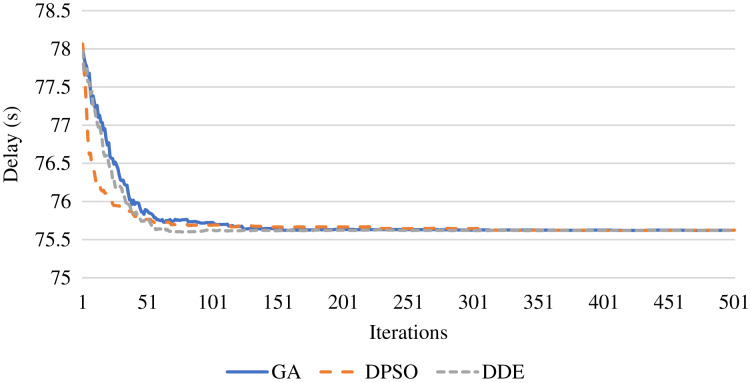
Optimization of the time delay using the DDE, PSO, and GA algorithms.

**Figure 6:**
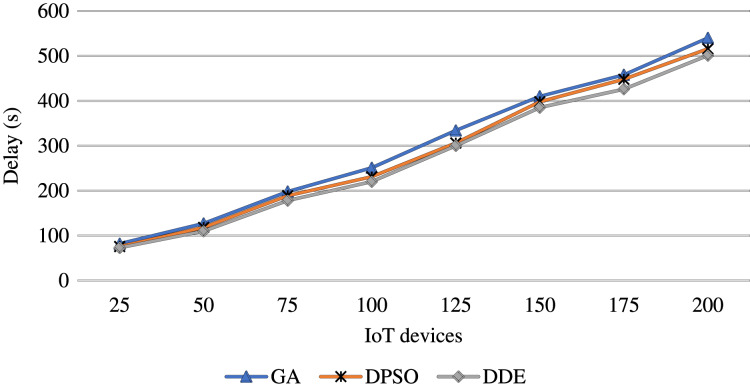
Average time delay obtained using the proposed meta-heuristics as increasing the IoT devices.

For the evaluation in terms of time delay, Fig. 5 shows the resulting delay during the iterations of the algorithms. The figure assures that the DDE algorithm is capable of identifying the lowest time delay faster than the DPSO and GA algorithms, which eﬀectively reduces the time delay. Further, Fig. 6 presents the average total time delay with an increasing number of IoT devices. This figure shows that the proposed strategy outperforms the comparison algorithm and successfully decreases the delay with an increasing number of oﬄoaded tasks.

**Figure 7:**
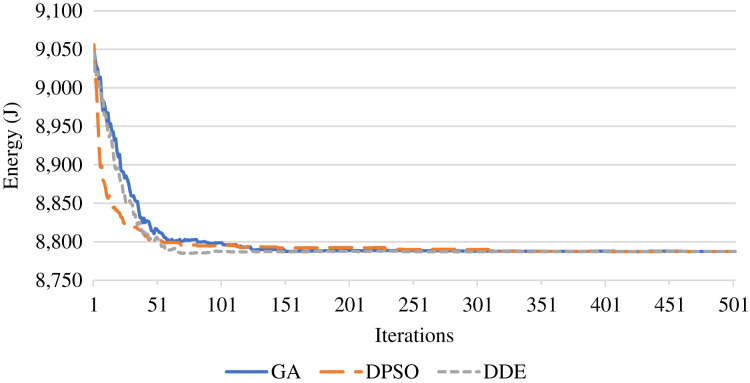
Optimization of the UAV energy using the DDE, PSO, and GA algorithms.

**Figure 8:**
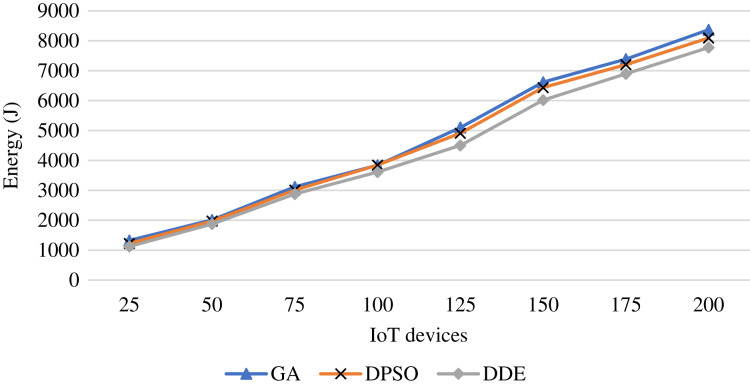
Average energy achieved using the proposed meta-heuristics as increasing the IoT devices.

In terms of energy, Fig. 7 shows the energy achieved during the iterations of a single run. The proposed meta-heuristics are capable of reducing the energy while the DDE algorithm maintains the lowest energy consumption. Further, Fig. 8 shows the average of the UAV energy as increasing the number of the IoT devices. This figure shows that as the number of IoT devices increases, the corresponding energy consumption increases. This increase is attributed to increasing the transmission and processing times, and consequently, the hovering time will increase. The DDE algorithm outperforms the PSO and GA algorithms and saves energy.

## Conclusion and future work

This paper proposed a UAV-based oﬄoading system that is aimed at minimizing the delay and the UAV energy consumption. Two diﬀerent subproblems for the proposed system are investigated. The first subproblem is the clustering of the IoT devices, where the proposed oﬄoading system partitions the ground devices into clusters, and the UAV hovers over each cluster head to process the oﬄoaded tasks for the cluster members. The second problem is the shortest path for the UAV to traverse the cluster heads. For the first sub-problem, two diﬀerent discrete meta-heuristic are proposed for the clustering process, namely, DDE and DPSO. The proposed meta-heuristics are compared with the GA. For the second subproblem, ACO is employed to identify the shortest path among the cluster heads. The experimental results show the eﬀectiveness of the proposed oﬄoading system using DDE and ACO in terms of delay, energy, the number of resulting clusters, and optimized trajectory of the UAV. Future work will consider the mobility of ground devices. We will investigate UAV-based, oﬄoading MEC with multi-cooperative UAVs to study the eﬀect of using multi-cooperative UAVs on the scope of the problem and the performance of the proposed strategy algorithm. Another interesting point for the future work is to test the proposed oﬄoading strategy on real-time tasks where the tasks execution time must meet a specified delay tolerance in the optimization, and to do a complete analysis on diﬀerent type of tasks, including compute-bound, network-bound, and real-time.

## Additional Information and Declarations

### Data Availability

The following information was supplied regarding data availability: The code is available at GitHub: https://github.com/mkhamiss/PeerJ.git.
